# Infiltrating ductal and lobular breast carcinomas are characterised by different interrelationships among markers related to angiogenesis and hormone dependence

**DOI:** 10.1038/sj.bjc.6600556

**Published:** 2002-11-04

**Authors:** D Coradini, C Pellizzaro, S Veneroni, L Ventura, M G Daidone

**Affiliations:** Department of Experimental Oncology, Determinants of Prognosis and Treatment Response Unit, Istituto Nazionale per lo Studio e la Cura dei Tumori, Milano, Italy

**Keywords:** lobular carcinoma, ductal carcinoma, angiogenesis, hormone responsiveness, apoptosis, proliferation

## Abstract

To obtain a more integrated understanding of the different breast cancer phenotypes and to investigate whether bio-molecular profiles can distinguish between specific histotypes, we explored the interrelations among several biologic variables indicative of, or related to, hormone dependence, proliferation and apoptosis control, and angiogenesis in ductal and lobular carcinomas, the most common histotypes. Oestrogen and progesterone receptors, tumour proliferative activity, the expression of cyclin A, p16^ink4A^, p27^kip1^, p21^waf1^, p53, bcl-2, and levels of vascular endothelial growth factor and hypoxia-inducible factor-1α (HIF-1α) were evaluated in 190 in ductal and 67 lobular carcinomas. Our findings support the hypothesis that in ductal and lobular carcinomas are two distinct, partially phenotypically unrelated entities, the latter being characterised by the presence of features indicative of differentiation such as oestrogen receptors, low proliferation and lack of p53 expression and associated with low vascular endothelial growth factor content compared to angiogenesis in ductal carcinomas. Conversely, no significant difference was found between lobular carcinomas and in ductal carcinomas considering the frequency distribution of PgR-positive cases, cyclin-dependent kinase inhibitors acting at the G1/S boundary, bcl-2 and HIF-1α protein expression. Although both generally defined as hormone responsive, in ductal and lobular carcinomas are also characterised by biologic patterns in which proteins related to hormone responsiveness, cell-cycle control, apoptosis and angiogenesis were differently associated. This finding suggests the need to refine breast cancer characterisation in order to provide detailed information about individual tumours, or subsets of tumours, that will help in defining optimal treatment approaches.

*British Journal of Cancer* (2002) **87**, 1105–1111. doi:10.1038/sj.bjc.6600556 www.bjcancer.com

© 2002 Cancer Research UK

## 

Despite its unique origin at the terminal duct–lobular unit (Lakhani *et al*, 1999) carcinoma of the breast is a very heterogeneous disease, due to the progressive gain or loss of functions accumulated during the expansion of the original transformed clone. In particular, infiltrating ductal (IDC) and lobular carcinomas (ILC) (the most frequently observed varieties of invasive breast cancer, accounting for 70–75% and 10–14% of all invasive tumours, respectively) are characterised by differences in their histological structures and natural history (Harris *et al*, 1992). In fact, even though following conservative surgery and radiation therapy patients with an ILC have an outcome similar to patients with IDC (Peiro *et al*, 2000), they show a different metastatic pattern, since bone marrow and peritoneum metastases are more prevalent in patients with ILC than in those with IDC (Borst and Ingold, 1993; Jain *et al*, 1993). ILC and IDC appear to be quite different, even in their biologic features. For instance, they differ in hormone receptor profile and proliferative activity – ILC are more frequently oestrogen (ER) and progesterone receptor (PgR) positive and slowly proliferating than IDC (Kruger *et al*, 1999). A different relation to angiogenesis has been recently shown and a lower expression of vascular endothelial growth factor (VEGF), the most selective and potent angiogenic factor till now identified (Ferrara, 1999) in ILC than in IDC has been described (Lee *et al*, 1998).

Translational studies have produced a large amount of biologic information on breast cancer. However, for the complex inter-relations among the cellular mechanisms involved in breast tumour growth and progression, this information has not always been evaluated as part of integrated pathways. In addition, most of these investigations focused almost exclusively on the ductal subtype or did not separately analyse the different histotypes. On the contrary, the analysis of bio-molecular profiles of the two histotypes, taking into consideration those biomarkers which are, singly, clinically relevant, could elucidate the distinctiveness of breast carcinoma subtypes and might provide information to understand specific metastatic patterns.

In an effort to obtain a more integrated understanding of the different breast cancer phenotypes, and to investigate whether bio-molecular profiles can distinguish between the two most common histotypes, we explored the inter-relations among several biologic variables (consolidated or of a more recent acquisition) related to hormone dependence (ER and PgR), proliferative activity potential (thymidine labelling index (TLI), cyclin A), cell-cycle and apoptosis control (p16^ink4A^, p27^kip1^, p21^waf1^, p53 and bcl-2) and angiogenesis (VEGF and hypoxia-inducible factor-1α (HIF-1α)) in IDC and ILC.

## MATERIALS AND METHODS

We studied 257 primary invasive breast carcinomas: 190 IDC and 67 ILC (pure or as main histotype in association to ductal histotype) obtained from patients submitted to surgery from May 1991 to May 1994 at the Istituto Nazionale Tumori of Milan. IDC and ILC were matched for patient age (median age: 57 years for both IDC and ILC), tumour size (median diameter: 1.9 and 2.0 for IDC and ILC, respectively) and axillary lymph nodes involvement (presence of node-positive cases, 21 and 18%, respectively). Fresh tumour material was obtained immediately after surgery. The tumour specimen was in part incubated with ^3^H-thymidine, fixed in neutral 10% buffered formaldehyde and then processed for conventional histological procedures for proliferative activity determination according to Silvestrini *et al* (1994) and for the *in situ* evaluation of the expression of p53, bcl-2, p16^ink4A^, p21^waf1^, p27^kip1^ and cyclin A, in part frozen in liquid nitrogen and stored at −80°C for ER and PgR detection by ligand-binding assay, as previously described (Ronchi *et al*, 1986), and for the determination of VEGF and HIF-1α levels (Table 1

**Table 1:**
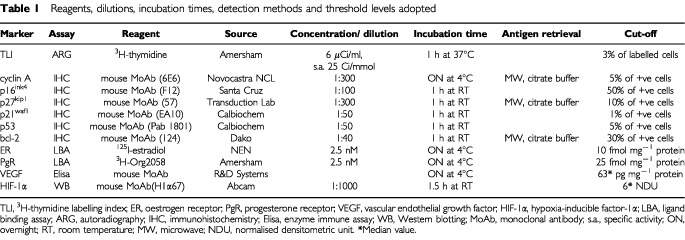
Reagents, dilutions, incubation times, detection methods and threshold levels adopted

). The determination of proliferation index and ER or PgR was performed within National Quality Control Programs (Piffanelli *et al*, 1989; Silvestrini, 1991) recently activated in Italy, also for p53 and bcl-2 expression (Paradiso *et al*, manuscript in preparation).

### *In situ* determinations

Proliferative activity was evaluated as the fraction of ^3^H-thymidine incorporating cells and was expressed as ^3^H-thymidine labelling index (TLI) as previously described (Silvestrini *et al*, 1994). The expression of p53 and bcl-2 was evaluated by immunohistochemistry on 4-μm–thick, paraffin-embedded sections as described elsewhere (Silvestrini *et al*, 1994). Expression of the other cell cycle-related proteins was immunohistochemically detected using monoclonal antibodies against p16^ink4A^ (Santa Cruz Biotechnology, Santa Cruz, CA, USA), p21^waf1^ (Calbiochem, La Jolla, CA, USA), p27^kip1^ (BD Transduction Laboratories, San Diego, CA, USA), and cyclin A (Novocastra Laboratories Ltd, Newcastle upon Tyne, UK) with dilutions, incubation times and procedures described in Table 1. Sections were processed for the avidin–biotin–peroxidase method (Vectastain ABC kit; Vector Laboratories, Burlingame, CA, USA). Negative controls were obtained by omission of primary antibodies, and tumours previously characterised by a high expression of each antigen were used as positive controls. The fraction of tumour cells positive at a cytoplasmic (for bcl-2), cytoplasmic and nuclear (for p16^ink4A^), or at a nuclear level (for the other antigens) was evaluated independently by two observers by scoring a total of 1000–3000 tumour cells and was defined as the percentage ratio between positive and total number of tumour cells.

### HIF-1*α* and VEGF determinations

HIF-1α and VEGF expression was evaluated on tumour nuclear and cytosolic fractions stored in our frozen bank following ER and PgR determination. VEGF was measured by a quantitative enzyme immunoassay technique (Quantikine human VEGF, R&D Systems, Minneapolis, MN, USA) according to manufacturer's instructions and expressed as pg of VEGF protein per mg of cytosolic protein (Coradini *et al*, 2001). HIF-1α levels were determined by Western blot analysis using the commercially available monoclonal antibody (H1α67, Abcam, Cambridge, UK). β-Actin was used as an internal control and was detected with a specific monoclonal antibody (Sigma Chemical, St. Louis, MO, USA). MCF7, a human breast cancer cell line that overexpresses HIF-1α, was used as a positive control. Immune complexes were detected using the ECL chemoluminescent system, and bands were detected by a ScanJet IIIcx/T, quantified by TotalLab software (Nonlinear Dynamics, Durham, NC, USA), normalised according to the internal standard, and expressed as normalised densitometric units (NDU). Before using actin bands to normalise the results, the presence of a linear range of protein concentration (i.e., a proportional increase in the actin signal by increasing the amount of starting protein concentration) was verified.

### Statistical analysis

The cut-off values (Table 1) used to discriminate between slowly or rapidly proliferating tumours, with or without steroid receptors and expressing p53 or bcl-2, proved to be biologically and clinically relevant in previous studies on large series of primary breast cancers in different clinical situations (Silvestrini *et al*, 1994; Daidone *et al*, 1999, 2000a). For p16^ink4A^, p21^waf1^, p27^kip1^ and cyclin A, we used cut-off values that in preliminary analyses provided relevant prognostic information (Daidone *et al*, 2000b). For VEGF and HIF-1α median values were used as cut-off.

The chi-square test was used to assess the statistical significance of differences in the frequency distribution of tumour features considered as categorical variables, whereas the Kruskal–Wallis test and Spearman rank correlation coefficient analysis were applied to assess the association between pathobiologic variables. Since due to the large number of analysis performed a proportion of the significant results should be due to chance alone, Bonferroni correction was applied to take account of multiple comparisons. All *P* values were two sided.

## RESULTS

The overall series of 257 invasive breast cancers can be considered representative of the bio-profile of human breast cancers, for the presence of ER and PgR in 84 and 69% of the cases, respectively, the prevalence of cases not expressing p53 (about 81%), and the presence of a similar fraction of bcl-2-positive and negative cases, in agreement with published results on larger series of cases.

Compared to IDC (Table 2

**Table 2:**
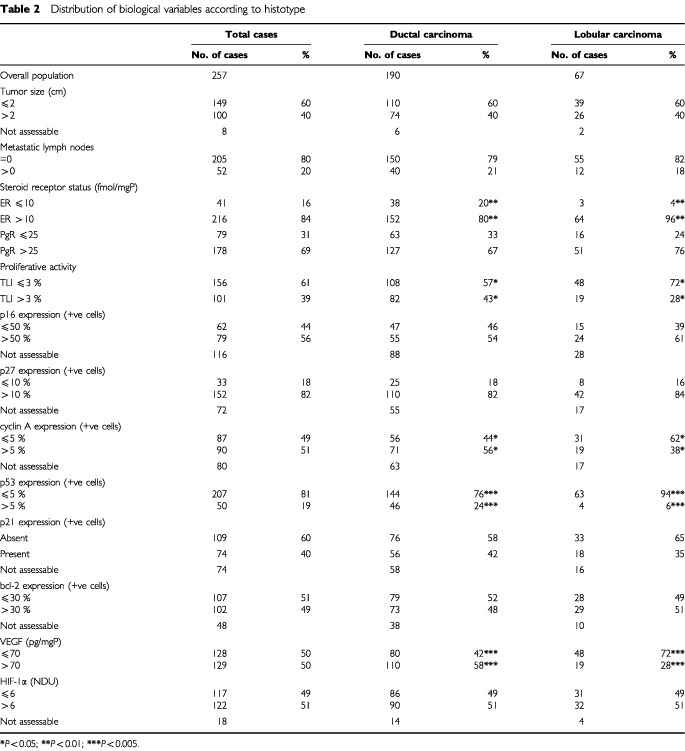
Distribution of biological variables according to histotype

), ILC were more frequently ER+ (96 *vs* 80%, *P*=0.003), slowly proliferating (72 *vs* 57%, *P*=0.03), p53-negative (94 *vs* 76%, *P*=0.001), cyclin A-negative (62 *vs* 44%, *P*=0.03) and VEGF-negative (72 *vs* 42%, *P*=0.001), with a statistically significant lower VEGF concentration (51 *vs* 91 pg mg^-1^ cytosolic protein, *P*=0.0001) or median value of cyclin A-positive cells (1.7 *vs* 6.2, *P*=0.008). Conversely, no difference was found between ILC and IDC considering the frequency distribution of PgR, p16^ink4A^, p27^kip1^, p21^waf1^, bcl-2 and HIF-1α protein expression.

Regardless of histotype, Spearman correlation coefficient analysis, adjusted by Bonferroni correction for multiple comparisons (Table 3

**Table 3:**
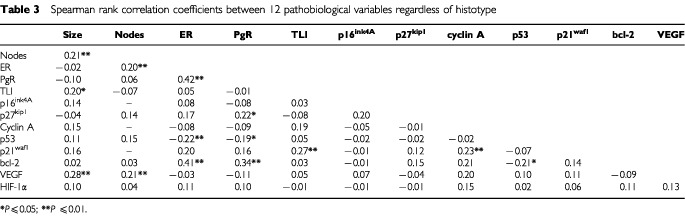
Spearman rank correlation coefficients between 12 pathobiological variables regardless of histotype

), indicated a statistically significant direct relation between ER and PgR (*P*=0.001), between ER or PgR and bcl-2 expression (*P*=0.001 in both cases), and between PgR and p27^kip1^ expression (*P*=0.03). Conversely, a statistically significant inverse relation was observed between ER, PgR or bcl-2 and p53 expression (*P*=0.004, *P*=0.02 and *P*=0.02, respectively). p21^waf1^ was directly related to TLI (*P*=0.002) and cyclin A expression (*P*=0.02). Within angiogenic variables, only VEGF showed a direct association with tumour size (*P*=0.001) and number of metastatic lymph nodes (*P*=0.009). p16^ink4A^ and HIF-1α proved to be unrelated to all the investigated variables, whereas a direct association was found between number of metastatic lymph nodes and tumour size or ER (*P*=0.008 and *P*=0.01, respectively) and between tumour size and TLI (*P*=0.05). However, it should be noted that, except for the association between ER and PgR, the correlation coefficients were generally low, thus indicating a weak association between variables.

When we analysed the relationships between biologic variables according to histotype (Tables 4

**Table 4:**
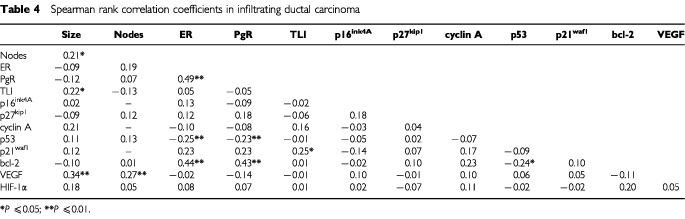
Spearman rank correlation coefficients in infiltrating ductal carcinoma

and 5

**Table 5:**
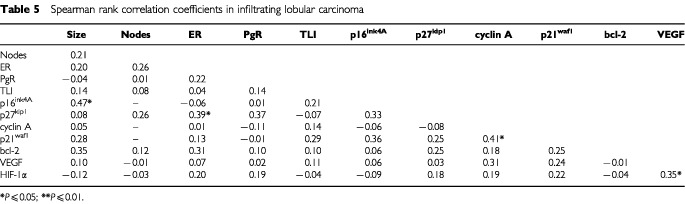
Spearman rank correlation coefficients in infiltrating lobular carcinoma

), a completely different profile of associations was observed. In fact, after eliminating weak correlations no relationship was shared by the two histotypes. A direct association between VEGF and tumour size (*P*=0.001) as well as between PgR and bcl-2 (*P*=0.001) were present only in IDC. In addition, tumour size was associated directly with TLI (*P*=0.02) only in IDC. Conversely, a direct correlation between p21^waf1^ and cyclin A (*P*=0.03), between p16^ink4A^ and tumour size (*P*=0.03), between VEGF and HIF-1α (*P*=0.05) and between p27^kip1^ and ER (*P*=0.05) was present only in ILC.

To investigate the presence of different profiles of association between biomarkers as a function of p53 expression, the relationships among biomarkers were investigated in IDC also within the subset of p53-negative tumours, which are comparable for p53 expression to ILC, which were p53-negative in the majority (63 out of 67 or 94%) of the cases. The direct relation between ER or PgR and bcl-2 (r_s_=0.38, *P*=0.001 and r_s_=0.40, *P*=0.001, respectively), VEGF and tumour size or number of metastatic lymph nodes (r_s_=0.33, *P*=0.001 and r_s_=0.27, *P*=0.01, respectively), TLI and p21^waf1^ (r_s_= 0.28, *P*=0.03) seemed to be independent of p53 expression, since they were observed in the subset of p53-negative IDC, as well as in the overall IDC series. Only a direct correlation between bcl-2 and cyclin A (r_s_=0.29, *P*=0.03) was present in p53-negative subset and not in the overall IDC series.

**Figure 1:**
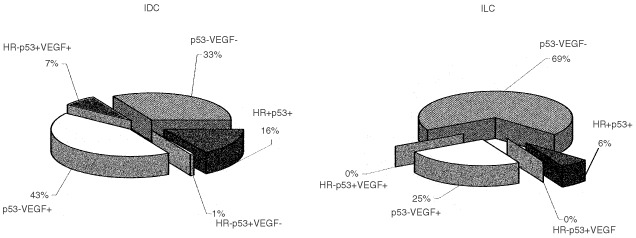
Graphical representation of the relationship among hormone responsiveness (HR), defined as the presence of at least one of the steroid receptors, cell-cycle control in terms of p53 presence and angiogenesis activation, in terms of VEGF concentration in infiltrating ductal (IDC) and lobular (ILC) carcinoma.

When we considered hormone responsiveness (defined as the presence of at least one of the hormone steroid receptors), cell-cycle control (as p53 expression) and angiogenesis activation (as VEGF protein level) (Figure 1

), we observed that the majority of ILC (69%) expressed a p53-negative and VEGF-negative phenotype, regardless of hormone receptor status, in comparison with 33% of IDC, while only a 25% of ILC were characterised by the p53-negative and VEGF-positive phenotype compared to 43% of IDC.

## DISCUSSION

The search for a better definition of breast carcinoma, in terms of phenotypic portrait, led us to explore the complex interrelationships existing among a panel of biologic variables representative of cellular mechanisms involved in proliferation, apoptosis and angiogenesis and related to tumour progression in IDC and ILC, the two main types of invasive breast cancer. The present results indicate that IDC and ILC, although both defined as hormone-responsive tumours (ER+ and/or PgR+ in 84 and 97% of cases, respectively), are characterised by a different biological profile in which proteins related to hormone responsiveness, cell-cycle control, apoptosis and angiogenesis are differently integrated. Such findings are in keeping with preliminary results obtained by the analysis of gene expression profiles of six ILCs and six IDCs (Zhao *et al*, 2002). As a whole, considering the association among three of the four biological markers exhibiting the most different pattern of expression between the two histotypes, namely steroid receptors, p53 and VEGF, ILC are characterised by the absence of p53 expression (thus including wild-type p53 cells but also p53 null cells) associated with very low VEGF levels in the majority of cases, regardless of hormone receptor status. Conversely, in IDC VEGF is expressed at high levels in about 60% of the cases and steroid receptors are preferentially related to the bcl-2 pathway, in agreement with previous results from Ioachim *et al* (2000), rather than to cyclins and cyclin-dependent kinase inhibitors pathway. Conversely, only an inverse relation between PgR and p53 was observed, thereby suggesting that the effect of PgR on cell differentiation might be prevalently exerted through the down-regulation of p53 expression and the up-regulation of bcl-2 expression. Although the bcl-2 protein prevents apoptosis and in pre-invasive lesions possibly promotes tumour development, in invasive carcinomas alterations in the extremely complex mechanism regulating apoptosis may occur and apoptosis-regulating proteins can be differently expressed and modulated in the different cellular context. Specifically, our results of a correlation between bcl-2 overexpression and biological features of a differentiated phenotype (high ER and PgR content and absence of p53 expression) in IDC suggest that bcl-2 is under hormonal control and could explain the reason why apoptosis-regulating proteins may be irrelevant to cell death (Blagosklonny, 2001) and associated to a more favourable clinical outcome (Silvestrini *et al*, 1994).

In ILC, despite the higher ER+ percentage and ER median content than IDC, ER was related only to p27^kip1^ expression. Differently from IDC, in which tumour size and number of metastatic lymph nodes were associated with VEGF expression, tumour growth and nodal involvement in ILC seemed to be independent of VEGF but related to the expression of other factors. In fact, a direct relation between tumour size (considered as a surrogate marker of progression) and p16^ink4A^ was found, thereby suggesting a link between an aberrant accumulation of these proteins with concurrent loss of their function and tumour proliferation (Emig *et al*, 1998).

As regards the angiogenesis-related proteins, we found that, as expected (Lee *et al*, 1998), IDC had a higher median VEGF concentration than ILC. In addition, a strong direct relation between VEGF content and IDC size or metastatic lymph nodes was observed. Conversely, in ILC, median VEGF concentration was very similar to that found in a series of normal breast tissues (43 pg mg^−1^ of cytosolic protein, data not shown) and an association between VEGF and HIF-1α was observed. These findings suggest that, in ILC, angiogenesis activation, although essential for tumour growth and progression, does not need VEGF overexpression but should be promoted through angiogenic factors others than VEGF, which might be HIF-1α-dependent. Otherwise, the presence of anti-angiogenic factors could also be hypothesised, as recently demonstrated in the myoepithelial cells surrounding lobular structures (Nguyen *et al*, 2000).

Taking into account the link found in IDC between steroid receptors and p53 and the high VEGF expression, the observation that, in our case series, the p53+/VEGF+ phenotype is more frequently associated with a negative steroid receptor status confirms previous findings. Moreover, in ILC the absence of a relation among steroid receptors, p53 and VEGF expression is not surprising. Clinical studies have demonstrated a significant relation between p53 status and VEGF expression and their correlation with a poor prognosis (Linderholm *et al*, 2000, 2001). The inter-relations among hormonal control, p53 and angiogenesis activation could be explained by the presence in the VEGF promoter region (Zhang *et al*, 2000) either of a specific cis-element recognised by p53 or of a specific oestrogen-response element (Hyder *et al*, 2000). In addition, p53 protein, less frequently mutated in ILC than in IDC (Marchetti *et al*, 1993), is able to down-regulate the promoter activity of VEGF in a dose-dependent manner (Mukhopadhyay *et al*, 1995) and to inactivate the HIF-1α protein (Blagosklonny *et al*, 1998). Conversely, the higher incidence of p53+ IDC compared to ILC, associated with a higher VEGF concentration, is in keeping with a possible down-regulation of angiogenesis by p53 tumour suppressor gene function through a block of HIF-1 activity, with the formation of a stable complex wild-type p53/HIF-1α (An *et al*, 1998) and the subsequent ubiquitination of HIF-1α. Mutation of the p53 gene and the generation of a nonfunctional protein that accumulates in the cell both inhibit the formation of the complex and result in the constitutive availability of the transcription factor and the subsequent transactivation of the VEGF gene (Ravi *et al*, 2000).

The present results provide evidence of the complex interrelationships among histological subtypes, hormone responsiveness, tumour growth, apoptosis and angiogenesis activation. They suggest the need for a refining of breast cancer characteristics in order to provide detailed information about individual tumours or subsets of morphologically similar cancers and to find a link between morphology, protein expression profile and biological behaviour. In this perspective, the advances in molecular biotechnologies, as recently demonstrated by Perou *et al* (2000), could supply information on alterations able to identify the genetic evolutionary pathways and explain the phenotypic differences observed in clinical tumours.
